# A cross-sectional analysis of U=U as a potential educative Intervention to mitigate HIV stigma among youth living with HIV in South Africa

**DOI:** 10.11604/pamj.2022.41.248.33079

**Published:** 2022-03-25

**Authors:** Israel Agaku, Lungile Nkosi, Joy Ngodoo Gwar, Tina Tsafa

**Affiliations:** 1 Department of Oral Health Policy and Epidemiology, Harvard School of Dental Medicine, Boston, MA, United States,; 2 Sefako Makgatho Health Sciences University (SMU), Pretoria, South Africa,; 3 Federal Medical Centre, Makurdi, Benue State, Nigeria,; 4 Department of Mass Communication, Benue State University, Makurdi, Nigeria

**Keywords:** Undetectable Equals Untransmissible (U=U), HIV/AIDS, HIV stigma, HIV testing

## Abstract

**Introduction:**

the HIV educative campaign Undetectable Equals Untransmissible (U=U) is a potential gamechanger to address HIV stigma. We investigated what percentage of South African adolescents were aware of U=U, and the associations with perceived HIV stigma and past-year HIV testing.

**Methods:**

we used a cross-sectional design. Data were from the 2017/2018 South African National HIV prevalence, incidence, behaviour and communication survey. HIV status was measured using both laboratory confirmation and self-reports. Among adolescents aged 15-18 years, we calculated the percentage who believed that “the risk of HIV transmission through sex can be reduced by an HIV-positive partner consistently taking drugs that treat HIV.” Data were weighted to yield nationally representative estimates.

**Results:**

overall, 49.8% of all adolescents aged 15-18 years (and 49.2% of this HIV seropositive) believed that the risk of HIV transmission through sex can be reduced by an HIV-positive partner consistently taking drugs that treat HIV. After adjusting for HIV status, geographic location, race, sex, and orphanhood status, those with belief in U=U were less likely to endorse stigmatizing statements that teachers with HIV should not teach (IRR=0.63, 95%CI, 0.47-0.84), pupils with HIV should not attend class (IRR=0.62, 95%CI, 0.45-0.84), or that children with HIV in general should be in segregated schools (IRR=0.55, 95%CI, 0.41-0.74). Among those reporting not living with HIV, U=U belief was associated with increased likelihood of past-year HIV testing (IRR=1.19, 95%CI, 1.01-1.41).

**Conclusion:**

U=U belief was associated with reduced stigma perceptions and increased HIV testing. Adoption of U=U into clinical practice guidelines in South Africa may benefit public health.

## Introduction

South Africa makes up less than 1% of the world´s population but contributed 18.9% (340,000) of the world´s 1.8 million children aged 0-14 years living with HIV in 2019; [1,2] 10,000 of these contributed cases were new ones from 2019 alone [1,3]. Despite availability of antiretroviral therapy (ART), 6,740 South African children aged 0-14 years died from HIV/AIDS in 2017 [1]. The impact of HIV among children and youth extends beyond those living with the disease; orphaned children from AIDS are also profoundly affected by stigma and other psychosocial challenges even if they do not have HIV. In 2016, there were 1.71 million South African children aged ≤17 years who had been orphaned by AIDS [1]. Conceivably, the psychosocial challenges experienced by children who acquired HIV at birth may differ markedly from adults who acquired it later in life, including the extent to which they internalize stigma [4]. Stigma among people living with HIV may be internalized (acceptance of negative HIV stereotypes), anticipated (fear of being discriminated against), or enacted (external stigma) [5]. Stigma exposure in the early formative years may impact youth living with HIV in ways that are profound, long-term, and consequential, and might perpetuate onward HIV transmission [6-9]. Fear of social rejection by friends/family/community, victimization, and negative attitudes from healthcare providers, have discouraged some from accessing HIV testing, adhering to HIV treatment, or utilizing health services in general [10,11]. Gender inequality is closely linked with HIV stigma as girls and young women are hardest hit by stigma and bear the greater burden of disease, accounting for 37% of new infections in 2016 [7]. Factors responsible for HIV infections among adolescent girls living with HIV include gender-based violence, sexual coercion, gender discrimination, multiple partners, and intergenerational/transactional relationships; limited condom use/challenges negotiating use; alcohol and substance abuse, poverty, poor nutrition, unemployment, and migration, and inadequate access to quality education [9,12].

Without addressing stigma, it would be challenging to meet the 2030 target of eradicating AIDS as a public health threat in South Africa, the country with the world´s largest HIV epidemic. Given that HIV stigma is largely driven by ignorance of the disease, its transmission, and the effects of treatment as prevention, the educational campaign known as Undetectable Equals Untransmissible (U=U) has emerged as a potential gamechanger. Based on a solid body of scientific research demonstrating that people living with HIV who have achieved and maintained ‘undetectability´ cannot transmit disease, [13-15] U=U has already been adopted into clinical practice guidelines in some countries but not yet in South Africa. To date, no study has examined youth awareness of U=U and the potential impact on HIV stigma in the South African context. Using nationally representative data, the study objectives were as follows: 1) to compare the emotional wellbeing of adolescents living with HIV with that of adults living with HIV as well as with their peers not living with HIV; 2) to examine the level of awareness of U=U among South African adolescents aged 15-18 years; 3) To examine the associations between U=U awareness and perceived HIV stigma and past-year HIV testing among 15-18-year-olds. We focused our analysis largely on the age group 15-18 years because this transitive period between childhood and adulthood is one where most South African youth initiate sexual activity [16,17]; the importance of promoting a culture of prevention at this critical stage cannot be overemphasized. More so, only teens were asked questions regarding current school enrollment and orphanhood status - important covariates in our analysis, especially as some of our study endpoints explored perceived stigma within the school context (e.g., stigma at fellow students or teachers with HIV). Preteens and younger adolescents aged <15 years were not part of the sampling frame of the analyzed survey component (i.e. adult) and were therefore not included.

## Methods

**Study design:** this was a cross-sectional study of South African households from the fifth cycle of the South African National HIV Prevalence, Incidence, Behaviour and Communication Survey (SABSSMV) [18].

**Study setting and population:** SABSSM-V is an ongoing, household-based survey that uses a multi-stage sampling process to select a nationally representative sample from the civilian, non-institutionalized South African population aged 0+ years. The adult component of the survey comprises persons aged 15 years or older. All individuals living in a household (including hostels) were eligible (response rate=82.2%). Electronic tablets were used for data collection. Consent was provided by all adult respondents and parental consent was obtained for minors <18 years.

**Sample size:** when estimating the sample size for the 2017 cycle, response rates were assumed to be similar to the historical baseline (84.7% for the household response rate, 89.5% for the individual response rate, and 67.5% for the HIV testing response rate) [19]. The national distribution of age, sex, race, locality/geotype and province formed the basis for generating the sample size for the 2017 survey national estimates. In total, 36, 609 individuals agreed to be interviewed; 61.1% of eligible participants provided a blood specimen for HIV-testing. These samples were anonymously linked to the completed questionnaires.

**The data collection tool:** four data collection tools from previous surveys were modified for the survey, [19-21] with inputs from various stakeholders. The tools were professionally translated from English into ten South African official languages [19]. For quality control, the translated tools were back translated into English by employees from the human sciences research council who were proficient in the various official languages. The data collection tools were then pretested among respondents from 150 households from ten small area layers (SALs) located in two provinces (Kwa-Zulu Natal and North West). The SALs were drawn from the 2015 national population sampling frame developed by Statistics South Africa (StatsSA).

### Variables

**HIV status, self-rated health, and stigma perceptions:** HIV status was ascertained using both laboratory assessment and self-report. Ever (≤1 time in lifetime) and past-year (≤1 time in the past 12 months) HIV testing was also assessed. Participants who rated their health as “fair”/“poor” (vs “excellent”/“good”) were classified as having suboptimal overall health. The survey also assessed past-30-day feelings of “tired out for no reason”, “nervous”, “so nervous that nothing could calm you down”, “hopeless”, “restless or fidgety”, “so restless you could not sit still”, “depressed”, “everything was an effort”, “so sad that nothing could cheer you up”, and “worthless”. Perceived stigma indicators were: “Would you want to keep the HIV-positive status of a family member a secret?”; “If a teacher has HIV but is not sick, should he or she be allowed to continue teaching?”; “If a pupil has HIV but not sick, should he or she be allowed to continue to go to school?”; “Do you think children living with HIV should be able to attend school with children who are HIV-negative?” Inaccurate information on HIV transmission was assessed with the question “Can a person get HIV by sharing food with someone who is infected?”

**U=U-related beliefs:** we were interested in exploring to what extent adolescents were aware that virally suppressed people living with HIV could enjoy good quality of life, including sexual intimacy without fear of HIV transmission to serodiscordant partners, or giving birth to children without HIV. In the survey, participants were asked to indicate if they believed that “the risk of HIV transmission through sex can be reduced by an HIV-positive partner consistently taking drugs that treat HIV” and that “a woman infected with HIV can have an HIV-negative baby”.

### Statistical analysis

**Descriptive and bivariate analysis:** weighted prevalence estimates were calculated for HIV seropositivity, perceived HIV stigma and U=U awareness among adolescents aged 15-18 years. Because of how intertwined HIV stigma and gender are, we explored when differences in HIV seroprevalence were most divergent between the genders across the lifespan. Furthermore, under our hypothesis that adolescents living with HIV may have the worst psychosocial challenges of any group, we compared indicators of self-rated mental and overall health between adolescents aged 15-18 years with HIV (n=60) vs adults aged >18 years with HIV (n=1916), as well as between adolescents aged 15-18 years without HIV (n=4089) vs adults aged >18 years without HIV (n=20,434) (all self-reported HIV statuses). Comparisons were performed with Chi-squared tests (p<0.05).

**Multivariable analysis:** to measure the associations between U=U awareness and perceived stigma among 15-18-year-olds, we pooled data of those with and without HIV to increase sample size, adjusting for HIV status, geographic location, race, sex, and orphanhood status. Adjusted prevalence ratios were calculated in a poisson regression model. Among 15-18-year-olds who reported not living with HIV, we measured the relationship between U=U awareness and past-year HIV testing, adjusting for geographic location, race, sex, and orphanhood status. All analyses were performed in Stata V14.

**Ethical considerations:** all analyses in this paper were done on secondary, de-identified, publicly available datasets. Ethical approval was not sought as the study was deemed to be non-human subject research.

## Results

**Sociodemographic characteristics and other descriptive statistics:** of South African adolescents aged 15-18 years, 83.4% were black, 51.0% male, 61.7% urban residents, 22.6% reported ≥one past-year sexual partner, and 25.9% reported ≥one biological parent dead (Table 1). The provinces with the highest youth distribution included KwaZulu-Natal (23.1%), Gauteng (21.6%), and Limpopo (12.2%).

**Table 1:** characteristics of the youth population aged 15-18 years, HIV seropositivity, perceived stigma, and awareness about indicators of U=U, South Africa, South African National HIV, Behaviour, and health survey, 2017/2018 (n = 4567)

Characteristics	% (n)	HIV seropositive	Believe a teacher with HIV should not be allowed to continue teaching even if they are not sick	Believe a pupil with HIV should not be allowed to continue to go to school even if they are not sick	Believe children living with HIV should not be able to attend school with children who are HIV-negative	Believe a person can get HIV by sharing food with someone who is infected	Would want to keep the HIV-positive status of a family member a secret	Believe that the risk of HIV transmission through sex can be reduced by an HIV-positive partner consistently taking drugs that treat HIV	Believe that a woman living with HIV can still have an HIV-negative baby
**Overall**	100.0 (4567)	4.5 (3.1-5.9)	13.8 (12.1-15.5)	12.9 (11.2-14.5)	13.2 (11.5-14.8)	14.1 (12.5-15.8)	69.9 (67.5-72.3)	49.8 (47.2-52.4)	65.0 (62.6-67.5)
**Province**									
Western Cape	10.6 (281)	1.3 (0.0-3.2)	24.7 (18.3-31.0)	18.4 (12.8-23.9)	18.2 (12.8-23.6)	13.2 (8.6-17.8)	62.1 (54.8-69.4)	49.4 (42.0-56.8)	61.9 (54.6-69.2)
Eastern Cape	10.0 (313)	6.7 (1.3-12.2)	12.9 (8.0-17.8)	17.3 (11.7-22.9)	19.2 (13.4-25.0)	13.1 (8.2-18.1)	62.8 (55.5-70.1)	35.7 (28.6-42.8)	57.7 (50.4-65.0)
Northern Cape	2.2 (216)	1.8 (0.0-3.7)	24.1 (16.4-31.9)	19.6 (12.4-26.8)	23.0 (15.3-30.6)	17.1 (10.1-24.1)	69.3 (61.4-77.2)	60.4 (51.8-69.0)	50.7 (42.1-59.3)
Free State	5.2 (175)	3.6 (0.0-7.1)	16.4 (9.7-23.1)	8.5 (3.6-13.4)	10.2 (4.8-15.6)	28.0 (19.6-36.4)	72.3 (64.3-80.2)	70.2 (62.2-78.3)	62.9 (54.2-71.6)
KwaZulu-Natal	23.1 (1779)	3.6 (1.4-5.7)	10.5 (7.4-13.7)	10.2 (7.3-13.0)	11.2 (8.1-14.2)	13.5 (10.2-16.8)	79.9 (76.0-83.8)	57.1 (52.4-61.9)	71.1 (66.7-75.5)
North-West	6.2 (245)	1.3 (0.0-3.1)	18.1 (12.2-24.0)	14.5 (9.1-19.9)	17.7 (11.7-23.7)	15.9 (10.2-21.5)	73.1 (66.3-80.0)	48.7 (41.0-56.5)	61.4 (53.8-69.0)
Gauteng	21.7 (566)	7.8 (3.4-12.3)	7.2 (3.6-10.9)	5.9 (2.5-9.3)	6.8 (3.2-10.4)	8.7 (4.6-12.9)	65.4 (58.4-72.3)	46.0 (38.7-53.3)	67.1 (60.2-74.0)
Mpumalanga	8.9 (681)	5.6 (0.4-10.7)	15.0 (10.2-19.7)	15.2 (10.5-20.0)	13.1 (8.7-17.6)	27.6 (21.7-33.5)	68.1 (61.9-74.3)	62.3 (55.8-68.8)	69.6 (63.5-75.7)
Limpopo	12.2 (292)	2.5 (0.3-4.7)	17.7 (11.8-23.5)	20.4 (14.2-26.5)	16.9 (11.1-22.7)	9.8 (5.5-14.1)	70.0 (63.3-76.7)	35.8 (28.9-42.7)	60.1 (52.9-67.2)
**Residence**									
Urban	61.7 (2217)	5.3 (3.2-7.4)	13.4 (11.1-15.6)	11.1 (9.0-13.2)	12.3 (10.1-14.4)	13.8 (11.6-16.1)	66.9 (63.5-70.2)	49.7 (46.2-53.2)	65.0 (61.6-68.3)
Rural informal	34.8 (1917)	3.2 (1.7-4.7)	13.7 (11.0-16.4)	15.2 (12.4-18.0)	13.6 (10.9-16.3)	14.2 (11.5-16.9)	75.2 (71.9-78.5)	49.8 (45.9-53.7)	66.4 (62.7-70.1)
Rural (farms)	3.6 (414)	2.9 (0.0-6.1)	22.9 (14.8-31.0)	20.2 (12.4-28.0)	24.6 (16.4-32.8)	19.2 (12.4-26.0)	70.2 (61.6-78.8)	51.1 (41.0-61.2)	52.8 (42.5-63.1)
**Race**									
Black African	83.4 (3786)	5.0 (3.4-6.6)	11.6 (9.8-13.3)	12.0 (10.3-13.7)	11.7 (10.0-13.4)	14.2 (12.3-16.1)	71.6 (69.0-74.2)	49.4 (46.5-52.2)	67.1 (64.4-69.8)
Non-Black	16.6 (751)	2.0 (0.0-4.9)	25.7 (20.3-31.1)	17.3 (12.7-21.9)	20.9 (16.0-25.8)	13.9 (10.2-17.7)	60.8 (54.8-66.7)	52.0 (46.0-57.9)	54.1 (48.2-60.1)
**Gender**									
Male	51.0 (2208)	4.8 (2.5-7.1)	15.4 (12.9-17.9)	13.4 (11.0-15.7)	14.5 (12.1-17.0)	15.8 (13.2-18.4)	70.7 (67.4-74.1)	50.2 (46.5-53.9)	58.9 (55.2-62.5)
Female	49.1 (2354)	4.2 (2.6-5.8)	12.2 (9.9-14.5)	12.3 (10.0-14.6)	11.8 (9.6-14.0)	12.5 (10.4-14.5)	69.0 (65.6-72.4)	49.3 (45.7-52.9)	71.4 (68.2-74.7)
**Orphanhood status**									
Both biological parents dead	7.1 (275)	9.9 (2.0-17.7)	14.1 (7.6-20.7)	12.4 (6.8-18.0)	16.4 (9.6-23.1)	11.8 (5.4-18.2)	71.2 (62.2-80.2)	46.8 (36.9-56.6)	69.5 (60.9-78.1)
One biological parent dead	18.9 (857)	8.6 (3.9-13.4)	12.4 (8.9-16.0)	10.3 (7.1-13.5)	12.3 (8.9-15.6)	15.7 (11.8-19.7)	68.0 (62.6-73.4)	49.5 (43.6-55.3)	64.3 (58.8-69.9)
Both biological parents alive	74.1 (3054)	2.7 (1.5-3.9)	14.1 (12.1-16.1)	13.5 (11.5-15.5)	13.1 (11.1-15.0)	14.0 (12.0-15.9)	70.2 (67.4-73.1)	50.1 (47.1-53.2)	64.8 (62.0-67.7)

**Figure 1:**
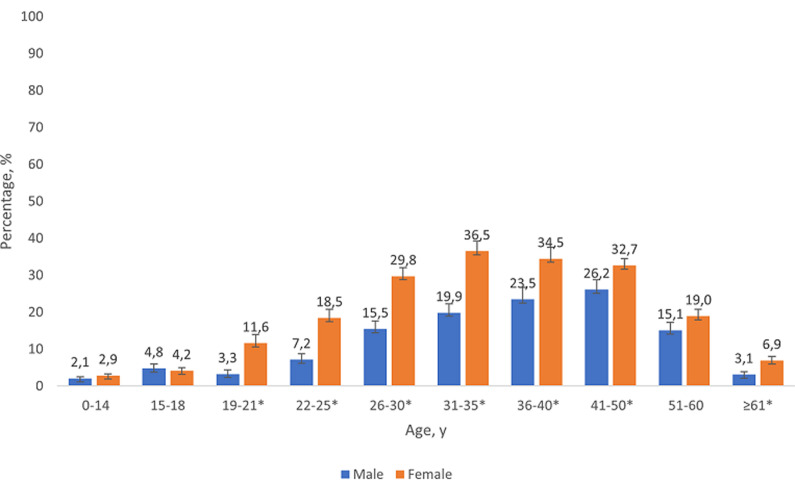
gender differences in HIV seropositivity across the lifespan, South Africa, South African National HIV, behaviour, and health survey, 2017/2018

**HIV Seropositivity, HIV testing, and self-rated health:** of all adolescents aged 15-18 years, 4.5% were HIV seropositive (151,220 individuals) (Table 1). HIV seroprevalence was highest in Gauteng (7.8%) and Eastern Cape (6.7%) provinces; urban areas (5.3%); among Black Africans (5.0%), males (4.8%), and those with either one (9.8%) or both biological parents dead (8.6%) vs none (2.7%). Examination of gender differences in HIV seropositivity across the lifespan revealed that no significant differences existed at ages 0-14 years (p=0.1631) or 15-18 years (p=0.646) (Figure 1). In all older age groups, except 51-60 years, female seroprevalence was significantly higher than male with the largest gap seen in the 19-21-year-old category (3.5-fold higher prevalence, 11.6% vs 3.3% respectively, p<0.001). Of HIV seropositive 15-18-year-olds, only 22.8% self-reported living with HIV; 86.6% however had either a report of living with HIV or an ART-related serum biomarker (i.e. ever diagnosed/treated). Sexual activity did not differ significantly between HIV seropositive vs negative adolescents, nor did other risk-taking behaviors such as injection drug use, ever drinking, or binge-drinking. Certain demographic factors however differed: a higher distribution of seropositive than negative adolescents resided in Gauteng (41.8% vs 22.1%), were Black Africans (96.3% vs 83.9%), and had lost ≥1 biological parent (50.9% vs 26.7%) (all p < 0.05).

**Figure 2:**
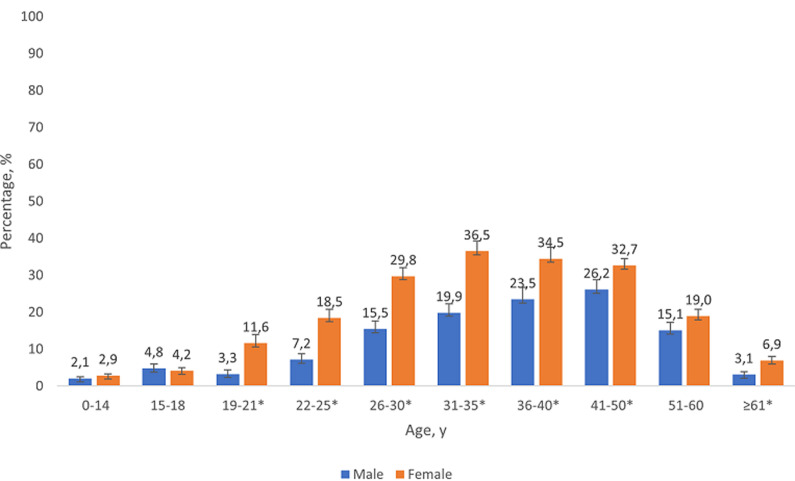
percentage who reported ever and past-year HIV testing among youth aged 15-18 years who reported not living with HIV (n = 4089)

Figure 2 shows prevalence of ever and past-year HIV testing by selected socio-demographics among adolescents aged 15-18 years who self-reported not living with HIV. Overall, only 37.5% of those self-reporting not living with HIV had ever had an HIV test and 26.4% reported a past-year HIV test. HIV testing, both ever and past-year respectively, were highest for Mpumalanga (52.9%, 40.8%), Black Africans (40.2%, 28.2%), ever had sexual intercourse (55.5%, 41.6%), had past-year sexual intercourse (56.3%, 42.4%), and rated their risk for HIV infection as high (48.1%, 34.3%, ever and past-year testing respectively). While no gender difference existed in ever HIV testing, females reported significantly higher prevalence of past-year HIV testing than males (29.8% vs 23.1%, p=0.004). Past-year testing was 42.6%, 42.5%, and 46.0% among those who reported engaging in past-year vaginal, anal, and oral sex respectively. Past-year HIV testing was also highest among those with one (42.4%) or 2+ past-year sexual partners (41.4%).

**Figure 3:**
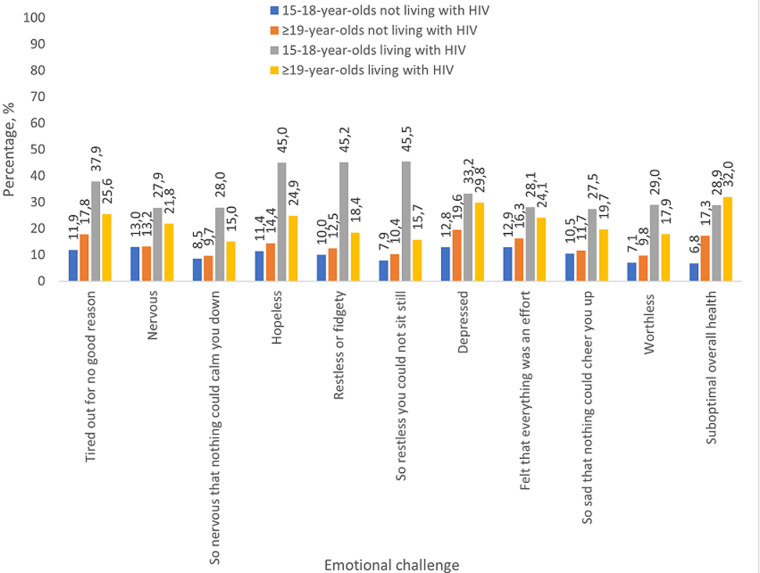
prevalence of self-rated mental and overall health indicators (%) comparing those reporting they were living with HIV ages 15-18 years (n = 60) and >18 years (n = 1916) as well as those reporting they were not living with HIV ages 15-18 years (n=4089) and >18 years (n =20,434)

A reversal was seen in age-specific self-rated mental health when comparing those with vs without a report of living with HIV (Figure 3). Among those reporting not living with HIV, adults aged >18 years reported worse emotional health than 15-18-year-olds; the reverse was seen among those reporting living with HIV. For example, the percentage reporting they felt “hopeless” among those reporting not living with HIV was 11.4% among 15-18-year-olds and 14.4% among >18-year-olds (p<0.01); among those reporting living with HIV, the corresponding percentages were 45.0% among 15-18-year-olds and 24.9% among >18-year-olds (p=0.047). Similar findings were also seen for other internalized negative emotions such as perceived worthlessness, sadness, depression, and being “tired out for no good reason”.

**U=U beliefs and associations with stigma:** overall, 49.8% of all adolescents aged 15-18 years (and 49.2% of this HIV seropositive) believed the risk of HIV transmission through sex can be reduced by an HIV-positive partner consistently taking drugs that treat HIV. Within the pooled analysis, this belief was highest among adolescents residing in Free State province (70.2%), and lowest among those in Eastern Cape (35.6%) and Limpopo (35.8%); no gender differences were observed (p=0.737). Furthermore, 65.0% overall (and 79.3% of this HIV seropositive) believed that a woman living with HIV could still give birth to an HIV negative baby, with prevalence higher among females than males (71.4% vs 58.8%, p<0.001). After adjusting for HIV status, geographic location, race, sex, and orphanhood status, adolescents who believed the risk of HIV transmission through sex can be reduced by an HIV-positive partner consistently taking drugs that treat HIV were less likely to endorse stigmatizing statements that teachers with HIV should not teach (IRR=0.63, 95%CI, 0.47-0.84), pupils with HIV should not attend class (IRR=0.62, 95%CI, 0.45-0.84), or that children with HIV in general should be in segregated schools (IRR=0.55, 95%CI, 0.41-0.74) (Table 2). Adolescents who believed women living with HIV could still give birth to HIV negative children were less likely to direct stigma at teachers with HIV (IRR=0.39, 95%CI, 0.29-0.52), at fellow students with HIV (IRR=0.32, 95%CI, 0.23-0.45), or to endorse segregation of HIV students (IRR=0.35, 95%CI, 0.25-0.48). Conversely, ignorance about how HIV is transmitted was associated with HIV stigma. Those believing HIV can be transmitted by sharing food were more likely to direct stigma at teachers with HIV (IRR=1.97, 95%CI, 1.45-2.67), at fellow students with HIV (IRR=1.96, 95%CI, 1.39-2.77), and to endorse segregation of HIV students (IRR=1.45, 95%CI, 1.02-2.06).

**Table 2:** associations between various indicators of accurate and inaccurate knowledge about HIV transmission and prevention, and markers of perceived stigma and HIV testing among South African youth aged 15-18 years, South Africa, South African National HIV, behaviour, and health survey, 2017/2018 (n = 4567)

Outcome	Exposure variable	Incidence rate ratio	P-value
Believe a teacher with HIV should not be allowed to continue teaching even if they are not sick (all participants)a	Believe that the risk of HIV transmission through sex can be reduced by an HIV-positive partner consistently taking drugs that treat HIV	0.63 (0.48-0.84)	0.002
	Believe that a woman living with HIV can still have an HIV-negative baby	0.39 (0.29-0.52)	<0.001
	Believe a person can get HIV by sharing food with someone who is infected	1.97 (1.45-2.67)	<0.001
Believe a pupil with HIV should not be allowed to continue to go to school even if they are not sick (all participants)a	Believe that the risk of HIV transmission through sex can be reduced by an HIV-positive partner consistently taking drugs that treat HIV	0.62 (0.45-0.84)	0.003
	Believe that a woman living with HIV can still have an HIV-negative baby	0.32 (0.23-0.45)	<0.001
	Believe a person can get HIV by sharing food with someone who is infected	1.96 (1.39-2.77)	<0.001
Believe children living with HIV should not be able to attend school with children who are HIV-negative (all participants)a	Believe that the risk of HIV transmission through sex can be reduced by an HIV-positive partner consistently taking drugs that treat HIV	0.55 (0.41-0.74)	<0.001
	Believe that a woman living with HIV can still have an HIV-negative baby	0.35 (0.25-0.48)	<0.001
	Believe a person can get HIV by sharing food with someone who is infected	1.45 (1.02-2.06)	0.04
Had HIV test in the past 12 months (participants reporting not living with HIV)b	Believe that the risk of HIV transmission through sex can be reduced by an HIV-positive partner consistently taking drugs that treat HIV	1.19 (1.00-1.41)	0.049
	Believe that a woman living with HIV can still have an HIV-negative baby	1.08 (0.90-1.30)	0.418
	Believe a person can get HIV by sharing food with someone who is infected	1.12 (0.90-1.39)	0.304

U=U beliefs were also associated with more HIV testing among those reporting they were not living with HIV. Among this sub-population of youth reporting not living with HIV, the likelihood of reporting a past-year HIV test was higher among those believing than not believing that the risk of HIV transmission through sex can be reduced by an HIV-positive partner consistently taking drugs that treat HIV (IRR=1.19, 95%CI, 1.01-1.41). Within adjusted analyses, past-year testing was higher among females than males (IRR=1.30, 95%CI, 1.09-1.55). Conversely, past-year testing was lower among non-black youth than black Africans (IRR=0.56, 95%CI, 0.43-0.73), those with both biological parents alive than those with both dead (IRR=0.68, 95%CI, 0.52-0.89), and those in rural informal areas than urban (IRR=0.81, 95%CI, 0.67-0.97).

## Discussion

Our findings suggest that the U=U educative intervention has potential to reduce HIV stigma, recalibrate the misperception that HIV is a death sentence, encourage early diagnosis, and incentivize engagement in care and ART adherence [15]. In counseling adolescents, it is important for pediatric practitioners to emphasize that U=U is about empowerment (especially with the high rates of perceived hopelessness among adolescents with HIV); it is not about condomless sex. Calabrese *et al*. [22] revealed that some health professionals have refrained from educating people living with HIV about U=U because of concerns that they will engage in more condomless sex or have more sexual partners upon learning of U=U. Withholding U=U education from people living with HIV is considered a violation of their sexual health and human rights and could potentially perpetuate onward HIV transmission [22]. Our results suggest that the social determinants that contribute to disproportionately higher HIV prevalence among females may be catalyzed during emerging adulthood, presumably when young women attain independence. No gender differences in HIV seroprevalence existed at ages 0-14 or 15-18 years, and the first (and largest) significant gap emerged at ages 19-21 years. Comprehensive strategies are therefore needed that address both gender inequalities and HIV inequalities as twin upstream determinants of stigma [23]. At the same time, continued education of the community through existing and new population-level communication campaigns (including U=U) can further reinforce preventive messages, ensure reach to broader audiences, and shift risk for the entire population. A 2012 evaluation by Peltzer *et al*. [24] showed high exposure to 18 different HIV communication programs among youth aged 15-24 years, with a significant association seen between exposure and greater HIV knowledge, condom use, past-year HIV testing, and reduced HIV stigma.

“Half knowledge” and misinterpretation of health messages are possible unintended consequences of health education campaigns. A related example is the campaign on male circumcision as a preventive measure against HIV, where “reduced HIV risk” became interpreted by some as “no HIV risk”. Many young South African females in one study inaccurately believed that circumcised men were 100% protected from HIV infection, [9] while most young males aged 10-19 in another [25] study assumed their HIV test was negative because they were circumcised. In the case of U=U, providers need to reinforce that U=U works, but only if people living with HIV are fully adherent and virally undetectable. Careful evaluation may be warranted for those with late diagnosis, or delayed treatment initiation who may have developed high pre-treatment viral loads that place them at risk for viral failure. As preventive measures, intensified efforts are needed to address myths, misconceptions, and unmet needs that contribute to people living with HIV not starting or not staying on treatment. Despite a plethora of mass media campaigns on the effectiveness of HIV treatment in South Africa [10], some adolescents still have doubts on the availability and effectiveness of HIV medication, a trend that could be a result of peer influence and lack of education/awareness. For those on treatment, improving retention in care is critical, so people can know their viral load and ascertain they are undetectable a key to empowering them and reversing internalized stigma [26]. The benefits of the U=U message may not only be limited to those living with HIV. In our study, awareness of U=U was associated with past-year HIV testing among those reporting not living with HIV. Intensified efforts to increase testing are needed as only a third of those reporting not living with HIV had ever tested, and even among those engaging in high-risk sexual activities such as anal sex, or having multiple sexual partners, less than half reported past-year testing. Reasons for poor adherence to HIV testing and treatment are not very different than those for poor adherence with other chronic medical conditions [27]. Barriers to HIV testing that have been reported in other studies include fear of receiving a positive HIV diagnosis, fear of being on treatment for a lifetime, anticipated stigma, aversion to being judged as “high-risk” or “immoral” by family/friends and poor mental outcomes such as depression and suicidal ideation [10,11]. Adoption of U=U into clinical practice guidelines in South Africa may accelerate the adoption and implementation in clinical settings beyond HIV treatment centers, which may help recalibrate social norms regarding HIV testing.

This study has certain limitations. First, the sampling frame excluded educational institutions, old-age homes, hospitals, homeless people, and uniformed-service barracks; these results may not be generalizable to these settings. Second, these are cross-sectional data, and no causal inferences can be made, only associations can be drawn. Third, self-reported data may be subject to misclassification and other social and cognitive biases. Fourth, there is a risk of omitted variable bias as we were unable to control for potential confounding factors of the relationship between U=U exposure and stigma perceptions. For example, health literacy/education in general (independent of U=U awareness in particular) may be associated with health-related perceptions; this was however not adjusted for in our study because of absence of validated indicators. However, we believe our findings to be robust despite this risk of bias because the analysis was restricted to a narrow age range (15-18 years) within which we expect to see little variability in the extent of health literacy/education; any resulting unmeasured confounding is therefore likely to be minimal. There may also be residual confounding as race was collapsed as all non-black racial groups were collapsed into one category because of sample size limitations. We encourage an exploration of these challenges in future research. Finally, we could not discriminate between perinatally infected youth and behaviorally infected youth because of lack of data. We encourage an exploration of these dimensions in future research.

## Conclusion

Overall, 49.8% of all adolescents aged 15-18 years (and 49.2% of those HIV seropositive) believed that the risk of HIV transmission through sex can be reduced by an HIV-positive partner consistently taking drugs that treat HIV. This belief was associated with reduced stigma perceptions among youth, as well as increased HIV testing among those reporting not living with HIV. Adoption of U=U into clinical practice guidelines in South Africa may benefit public health.

### What is known about this topic

- South Africa has the world´s largest number of youth living with HIV and in 2017, 6,740 South African children aged 0-14 years died from HIV/AIDS. HIV stigma is common and intricately tied with gender inequality and ignorance about HIV transmission/prevention.

### What this study adds

- Fourty-nine point eight percent of adolescents 15-18 years (and 49.2% of those HIV seropositive) reported U=U belief. U=U belief was associated with reduced stigma perceptions and increased HIV testing. Adoption of U=U into clinical practice guidelines in South Africa may benefit public health.
